# Plasma biomarkers increase diagnostic confidence in patients with Alzheimer’s disease or frontotemporal lobar degeneration

**DOI:** 10.1186/s13195-024-01474-z

**Published:** 2024-05-11

**Authors:** Daniele Altomare, Ilenia Libri, Antonella Alberici, Jasmine Rivolta, Alessandro Padovani, Nicholas J. Ashton, Henrik Zetterberg, Kaj Blennow, Barbara Borroni

**Affiliations:** 1 Department of Clinical and Experimental Sciences, Neurology Unit, University of Brescia, Brescia, Italy; 2 Department of Continuity of Care and Frailty, Azienda Socio Sanitaria Territoriale (ASST) Spedali Civili, Brescia, Italy; 3 Department of Psychiatry and Neurochemistry, Institute of Neuroscience and Physiology, the Sahlgrenska Academy at the University of Gothenburg, Mölndal, Sweden; 4 Institute of Psychiatry, Psychology and Neuroscience, King’s College London, Maurice Wohl Clinical Neuroscience Institute, London, UK; 5 NIHR Maudsley Biomedical Research Centre, South London and Maudsley NHS Foundation Trust, London, UK; 6 Centre for Age-Related Medicine, Stavanger University Hospital, Stavanger, Norway; 7 Clinical Neurochemistry Laboratory, Sahlgrenska University Hospital, Mölndal, Sweden; 8 Department of Neurodegenerative Disease, UCL Institute of Neurology, Queen Square, London, UK; 9 UK Dementia Research Institute, UCL, London, W1T 7NF UK; 10 Kong Center for Neurodegenerative Diseases, Clear Water Bay, Hong Kong, China

**Keywords:** Plasma biomarkers, Alzheimer’s disease, Frontotemporal lobar degeneration, Diagnosis, Diagnostic confidence

## Abstract

**Background:**

The recent development of techniques to assess plasma biomarkers has changed the way the research community envisions the future of diagnosis and management of Alzheimer’s disease (AD) and other neurodegenerative disorders. This work aims to provide real world evidence on the clinical impact of plasma biomarkers in an academic tertiary care center.

**Methods:**

Anonymized clinical reports of patients diagnosed with AD or Frontotemporal Lobar Degeneration with available plasma biomarkers (Aβ_42_, Aβ_42_/Aβ_40_, p-tau_181_, p-tau_231_, NfL, GFAP) were independently assessed by two neurologists who expressed diagnosis and diagnostic confidence three times: (T0) at baseline based on the information collected during the first visit, (T1) after plasma biomarkers, and (T2) after traditional biomarkers (when available). Finally, we assessed whether clinicians’ interpretation of plasma biomarkers and the consequent clinical impact are consistent with the final diagnosis, determined after the conclusion of the diagnostic clinical and instrumental work-up by the actual managing physicians who had complete access to all available information.

**Results:**

Clinicians assessed 122 reports, and their concordance ranged from 81 to 91% at the three time points. At T1, the presentation of plasma biomarkers resulted in a change of diagnosis in 2% (2/122, *p* = 1.00) of cases, and in increased diagnostic confidence in 76% (91/120, *p* < 0.001) of cases with confirmed diagnosis. The change in diagnosis and the increase in diagnostic confidence after plasma biomarkers were consistent with the final diagnosis in 100% (2/2) and 81% (74/91) of cases, respectively. At T2, the presentation of traditional biomarkers resulted in a further change of diagnosis in 13% (12/94, *p* = 0.149) of cases, and in increased diagnostic confidence in 88% (72/82, *p* < 0.001) of cases with confirmed diagnosis.

**Conclusions:**

In an academic tertiary care center, plasma biomarkers supported clinicians by increasing their diagnostic confidence in most cases, despite a negligible impact on diagnosis. Future prospective studies are needed to assess the full potential of plasma biomarkers on clinical grounds.

**Supplementary Information:**

The online version contains supplementary material available at 10.1186/s13195-024-01474-z.

## Background

Differential diagnosis in patients with cognitive impairment poses significant challenges due to the overlap in clinical symptoms. Cognitive impairment is often caused by neurodegenerative diseases which include Alzheimer’s disease (AD) and Frontotemporal Lobar Degeneration (FTLD). AD represents the most prevalent cause of dementia, accounting for 60–80% of dementia cases [1]. AD is characterized by the abnormal deposition of amyloid beta (Aβ) and tau proteins in the brain [2] which, together with other events such as reactive astrogliosis [3] and neurodegeneration, contribute to the onset of cognitive impairment and finally dementia. FTLD is a clinically and pathologically heterogeneous condition that results in progressive decline in behavior (behavioral variant of frontotemporal dementia, bvFTD) or language (primary progressive aphasia, PPA), and is often associated with extrapyramidal symptoms such as corticobasal syndrome (CBS) or progressive supranuclear palsy (PSP) [4].

In this context, biomarkers provide valuable information on the presence and burden of pathophysiological changes in the brain, and are therefore widely used in clinical practice (at least in academic settings) as part of the patients’ diagnostic workup to support the clinical diagnosis [5], and therefore to discriminate AD from other neurodegenerative disorders. The use of AD biomarkers will likely be more relevant in the next future as disease-modifying therapies are becoming available [6, 7]. However, the key AD biomarkers are traditionally measured with techniques/exams that are either expensive (amyloid-PET and tau-PET), invasive (cerebrospinal fluid (CSF) Aβ_42_/Aβ_40_ and p-tau), or poorly specific (atrophy on MRI and hypometabolism on FDG-PET).

The recent development of techniques allowing to measure biomarkers of AD pathology (Aβ_42_/Aβ_40 _[8] and p-tau [9–11]), neurodegeneration (NfL) [12], and astrogliosis (GFAP) [13] in plasma has changed the way the research community envisions the future of diagnosis and management of patients with suspected AD or other neurodegenerative disorders. Indeed, plasma biomarkers feature unique advantages over traditional biomarkers such as higher accessibility, relatively lower cost, and higher informativeness (providing information on several specific and non-specific AD biomarkers from a single blood sampling). Owing to these features, plasma biomarkers could significantly improve the efficiency of the diagnostic pathway in memory clinics and finally improve patient care. Their future potential applications are various, being used as a gateway to traditional biomarkers [14], diagnostic, monitoring of disease progression or efficacy of treatments, and even screening of the general population [15].

In the last five years, a number of studies have evaluated the diagnostic accuracy of plasma biomarkers in several populations, reporting an overall high accuracy at the group level [8–13]. It is worth noting that, despite the excellent diagnostic accuracy at the group level, their performance in the real world might be poorer [14]. Therefore, despite consistent evidence on their diagnostic accuracy, further research is needed to understand the role and performance of plasma biomarkers in clinical practice.

The present study aims to investigate the role of plasma biomarkers in the diagnostic thinking of clinicians in an academic tertiary care center. Specifically, we assess whether the use of plasma biomarkers as a first-level assessment impacts diagnosis and diagnostic confidence, and whether clinicians’ interpretation of plasma biomarkers and the consequent clinical impact are appropriate (i.e., consistent with the final diagnosis determined after the conclusion of the diagnostic work-up).

## Methods

### Study design

**Fig. 1:**
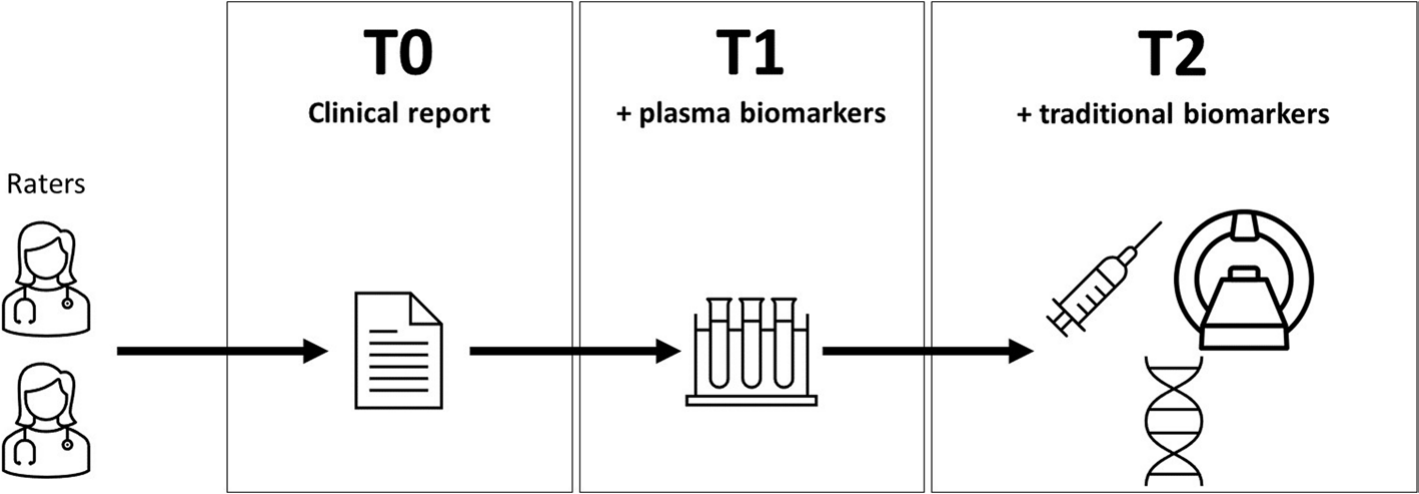
Study design. Two raters assessed the clinical reports of the patients at different time points: at T0, based on the clinical first-visit reports; at T1, based also on the plasma biomarker results; and at T2, based also on traditional biomarkers (when available). At each time point, raters were asked to indicate diagnosis and rate their diagnostic confidence (50–100%) in such diagnosis for each patient. Traditional biomarkers included cerebrospinal fluid analyses (CSF Aβ_42_, p-tau, and total tau), amyloid-PET scan, and genetic assessment

Figure 1 illustrates the study design of the present work. Two independent raters (AA and IL, both neurologists with 25 and 4 years of expertise in neurodegenerative disorders, respectively) blindly assessed the anonymized clinical reports of patients for whom plasma biomarkers were available at three time points.

At T0 (baseline), the two raters assessed the clinical reports including all the information collected during the first visit at our center, and were asked to indicate a cognitive stage (i.e., mild cognitive impairment (MCI) or dementia) and diagnosis (i.e., AD, bvFTD, PPA, CBS/PSP), and to rate their diagnostic confidence (50–100%) in such diagnosis. Specifically, the first-visit clinical reports included the patient’s demographics, past and present comorbidities, family history, description of the first symptoms at onset, global cognitive assessment, associated behavioral symptoms, and structural brain imaging in most cases. At T1, the two raters were asked to revise the baseline diagnosis and diagnostic confidence for each patient based on the plasma biomarker results. Specifically, raters had access to and interpreted the values of all available plasma biomarkers at the same time, together with a panel describing values (minimum, median and IQR, mean and SD, and maximum value as well as the distribution; see section e2.1, eTable, and eFigure in Supplement) of a sample of 27 cognitively unimpaired individuals (median ± IQR age: 48 ± 26 years; gender: 44% (12/27) of males) as a reference. Importantly, we did not provide thresholds defining positivity/negativity of the plasma biomarkers (but only the raw values to be interpreted comprehensively based on the values of cognitively unimpaired individuals). More information on material and preparatory activities is reported in section e2.1 in Supplement. Finally, at T2, the two raters were asked to revise diagnosis and diagnostic confidence based on the results of traditional exams such as CSF analyses, amyloid-PET scan, or genetic investigation. When the two raters were not concordant, a third rater (BB) was asked to assess these cases at all time points.

Finally, in order to assess whether raters’ interpretation of plasma biomarkers and the consequent clinical impact (i.e., changes in diagnosis and diagnostic confidence) were appropriate, we used (as the gold standard) the final diagnosis provided by the dementia experts who had the patients in charge and complete access to all available information such as the clinical and instrumental work-up (see "Participants" section). The final diagnosis was commonly achieved approximately 4 months after the first visit.

### Participants

The reports of 146 outpatients with cognitive impairment evaluated at the Neurology Unit of the University of Brescia and the ASST Spedali Civili (Brescia, Italy), for whom plasma biomarkers were quantified, were initially considered in the present study. Among them, 24 have been excluded (23 were not informative enough or inconclusive at the first visit report, and 1 was recognized by one rater despite anonymization), for a total of 122 reports included in the analyses. Final diagnosis was consistent with either AD, bvFTD, PPA, CBS, or PSP, according to conventional clinical criteria [16–23]. All included patients underwent a standardized neuropsychological evaluation and brain magnetic resonance imaging (MRI), as previously reported [24]. Furthermore, CSF analyses (i.e., Aβ_42_, p-tau, and t-tau) and amyloid PET scan were available in 57% (70/122) and 19% (23/122) of cases, respectively, to support or rule-out AD. Proof of pathogenetic mutations was available in 18% (22/122) of cases, supporting definitive diagnosis of FTD (*C9orf72* expansion, *n* = 6; *Granulin* mutations, *n* = 13; *Microtuble-Associated Protein Tau* mutations, *n* = 2; and *TAR DNA-binding protein 43* mutation, *n* = 1).

The study was approved by the local Ethic Committee (NP1965), and has been conducted in accordance with the principles of the Declaration of Helsinki and the International Conference on Harmonization Good Clinical Practice.

### Outcomes

The two primary outcomes were change in diagnosis and change in diagnostic confidence across time points in the whole sample. The diagnoses were categorized into three categories: (i) “AD”, including all the diagnoses involving AD; (ii) “FTD”, including bvFTD and PPA; and (iii) “CBS/PSP” including CBS and PSP. Diagnostic confidence was rated by ticking the percentage corresponding to their appraisal of the diagnostic confidence on a visual numeric scale made of percentages organized spatially from 50 to 100% with incremental 5%-intervals. Before the beginning of the study, the study team (including the raters) defined the following criteria to objectivize, as much as possible, the subjective nature of diagnostic confidence: (i) 50% corresponds to max uncertainty, (ii) 90% defines a “very high” diagnostic confidence as operationalized by previous studies [25], and consistently with previous studies showing that the etiological diagnosis of patients with a diagnostic confidence greater than 90% does not change following amyloid-PET [26], and that the maximal mean diagnostic confidence post amyloid-PET is 86–93% [27–30], suggesting that this level of diagnostic confidence is a strong, achievable, and replicable reference standard; and (iii) 100% corresponds to max certainty. For the analyses, we considered the diagnosis expressed in agreement by two concordant raters (i.e., the first two raters or, when they were discordant, one of them and the third one), and the average of their diagnostic confidence only for patients with a diagnosis confirmed across different time points.

### Assessment of plasma biomarkers

Plasma was collected at the first visit by venipuncture, processed and stored in aliquots at -80°C according to standardized procedures, and analyzed in a central laboratory. Specifically, plasma p-tau_181_ and p-tau_231_were analyzed using an in-house single-molecule array (Simoa) method developed at the University of Gothenburg [9, 10]. Aβ_42_/Aβ_40_, NfL, and GFAP were analyzed using a commercial Simoa multiplex assay [31–33]. Plasma samples were thawed, vortexed, and centrifuged (4000 × g for 10 min at RT), then analyzed by a HD-X analyzer using identical batches of reagents across the study. Three quality control plasma samples were added in duplicate to the test plates at the start and end of each run, resulting in an overall coefficient of variation of 4.9% to 12.5% across all the plasma marker measurements.

### Statistical analyses

Continuous variables were described as median and interquartile range (IQR), and categorical variables as percentages (raw numbers). Differences among groups in the sociodemographic and clinical features were assessed using Kruskal–Wallis rank sum tests for continuous variables, or tests for equality of proportions for categorical variables. If significant, post-hoc pairwise comparisons (Dunn’s all-pairs rank comparison test for continuous variables, or pairwise comparisons for categorical variables) were adjusted using Bonferroni correction.

The inter-rater agreement between the two raters (AA and IL) for the clustered diagnoses (AD and FTLD) at the different time points was assessed using the unweighted Cohen’s *k* coefficient, and strength of agreement classified as *slight* (0.00 – 0.20), *fair* (0.20 – 0.40), *moderate* (0.40 – 0.60), *good* (0.60 – 0.80), and *very good* (> 0.80), with 95% confidence intervals (CI).

Changes in diagnosis (from AD to FTLD) after plasma biomarkers were assessed using the McNemar's Chi-squared test (*χ*^2^). Changes in diagnostic confidence in patients with confirmed diagnosis across timepoints were assessed using a linear mixed model with diagnostic confidence as the dependent variable; diagnosis (AD, FTD, or CBS/PSP), time point (T0, T1, and T2), and their interaction as independent variables; and random intercepts and slopes at the subject level.

All statistical analyses were performed with R, version 4.3.0 (The R Project for Statistical Computing, https://www.r-project.org/).

## Results

### Participants’ features

A total of 122 cases were included in the analyses. Table 1 illustrates demographic and clinical features of the patients. On average, the study participants were 64 ± 13 years old, included 57% (70/122) of males, had 10 ± 5 years of education, and MMSE was 25 ± 5. Among them, 48% (58/122) were in the MCI stage. Disaggregating by final diagnosis, 24% (29/122) were AD, 40% (49/122) bvFTD, 25% (31/122) PPA, and 11% (13/122) CBS/PSP.

**Table 1:** Demographic and clinical features of the sample

Demographic and clinical features	Whole sample *n* = 122	By final diagnosis				
		**AD** ***n* = 29**	**FTLD**			***p*-value**
			**bvFTD** ***n* = 49**	**PPA** ***n* = 31**	**CBS/PSP** ***n* = 13**	
Age, years	64 (13)	65 (12)	61 (16)	64 (10)	65 (12)	0.159
Age at symptom onset, years	61 (12)	63 (13)	57 (14)	63 (8)	62 (7)	0.027^*^
Gender, male (%)	57% (70)	55% (16)	69% (34)	39% (12)	62% (8)	0.059
Education, years	10 (5) [4]	11 (6) [1]	8 (5) [1]	13 (8) [2]	13 (5)	0.172
MMSE	25 (5) [11]	24 (4) [2]	25 (6) [4]	26 (6) [5]	26 (6)	0.359
Cognitive stage, MCI (%)	48% (58)	45% (13)	45% (22)	45% (14)	69% (9)	0.432

Values are medians (interquartile ranges) for continuous variables, or percentages (raw numbers) for categorical variables

Diagnoses were based on the final diagnosis determined after the conclusion of the diagnostic clinical and instrumental work-up by the actual managing physicians who visited the patient in person. Cognitive stages were based on the consensual assessment of the study raters

*AD* Alzheimer’s disease, *bvFTD* behavioral variant of frontotemporal dementia, *CBS* corticobasal syndrome, *MCI* mild cognitive impairment, *MMSE* Mini-Mental State Examination, *PPA* primary progressive aphasia, *PSP* progressive supranuclear palsy

^*^No pairwise comparison survived the Bonferroni correction

[number in square brackets]: number of missing data

### Raters’ concordance

The inter-rater agreement on the diagnosis (AD or FTLD) was *moderate* at T0 (84% (103/122) concordance rate; unweighted *k* = 0.52, 95% CI 0.33 – 0.70) and T1 (81% (99/122) concordance rate; unweighted *k* = 0.46, 95% CI 0.27 – 0.65), but increased to *good* with traditional biomarker availability at T2 (91% (86/94) concordance rate; unweighted *k* = 0.78, 95% CI 0.64 – 0.92).

### Change in diagnosis

**Fig. 2:**
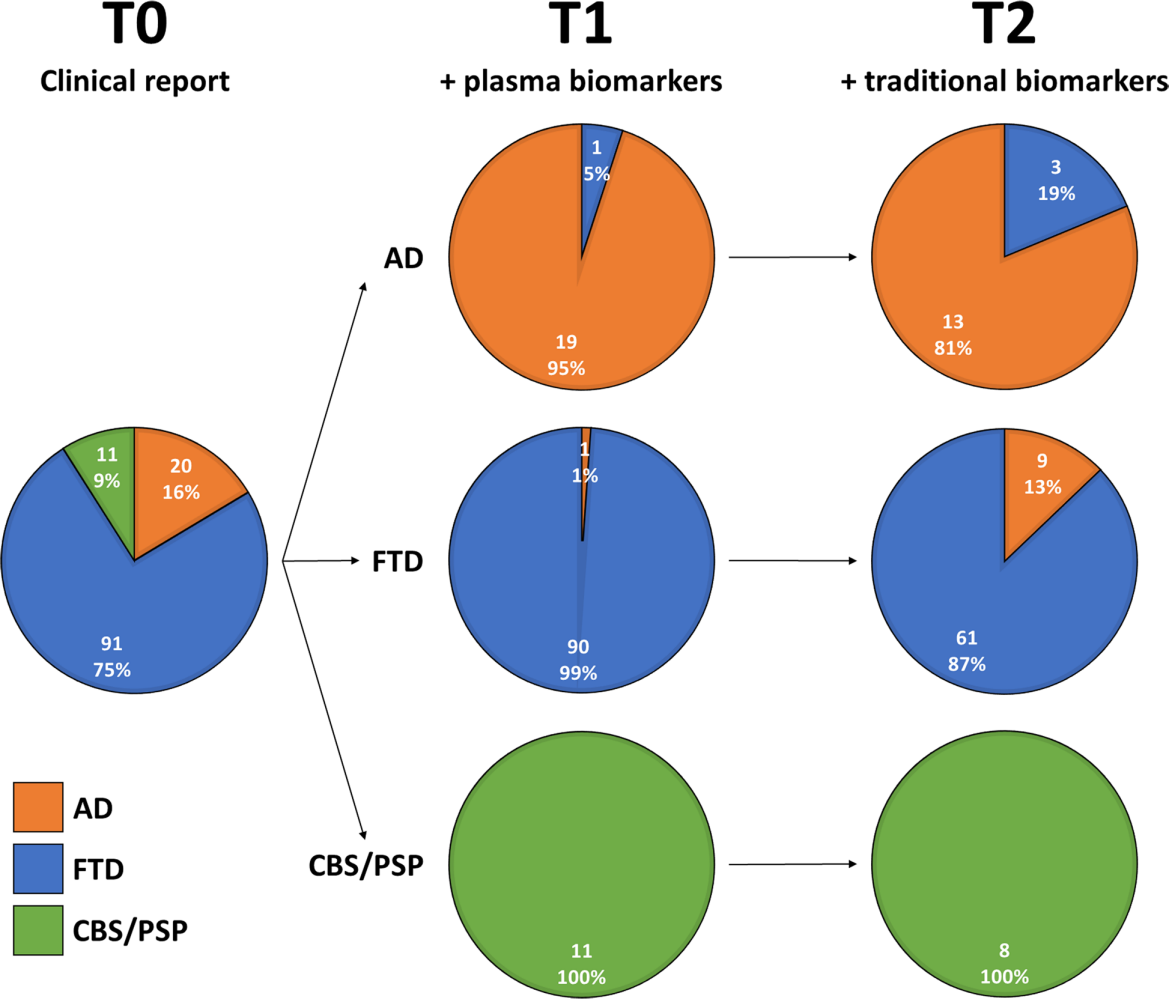
Change in diagnosis across time points. Reading example: at T0, 16% (20/122) of patients received a diagnosis of AD; among them, at T1, 5% (1/20) of patients with a T0 diagnosis of AD were reclassified as non-AD after plasma biomarkers; finally, at T2, in a sub-sample of patients for whom traditional biomarkers were available, 19% (3/16) of patients with a T1 diagnosis of AD were reclassified as FTD. Traditional biomarkers could include cerebrospinal fluid analyses (CSF Aβ_42_, p-tau_181_, and total tau), amyloid-PET, or genetic assessment. AD: Alzheimer’s disease. FTD: frontotemporal dementia. CBS: corticobasal syndrome. PSP: progressive supranuclear palsy

Figure 2 illustrates the change in diagnosis across the different time points.

At T1, the presentation of plasma biomarkers resulted in a change of diagnosis in 2% (2/122; *χ*^2^ = 0.00, *p* = 1.00) of patients. Specifically, diagnostic changes occurred in 1 patient who had a T0 diagnosis of AD that changed to FTD (bvFTD) at T1, and in 1 with a T0 diagnosis of FTD (PPA) that changed to AD at T1. In both cases (100%, 2/2), changes in diagnosis were consistent with the final diagnosis.

At T2, in a subgroup of patients (*n* = 94), the addition of the traditional biomarkers resulted in a further change of diagnosis in 13% (12/94; *χ*^2^ = 2.08, *p* = 0.149) of cases. Specifically, diagnostic changes occurred in 3 patients who had a T1 diagnosis of AD that changed to FTD (2 to bvFTD and 1 to PPA), and in 9 with a T1 diagnosis of FTD (5 bvFTD and 4 PPA) that changed to AD at T1. In 92% (11/12) of cases, changes in diagnosis were consistent with the final diagnosis.

Diagnosis changed more frequently at T2 than at T1 (*p* = 0.003), with a comparable consistency with the final diagnosis at T1 and T2 (*p* = 1.00).

### Change in diagnostic confidence

**Fig. 3:**
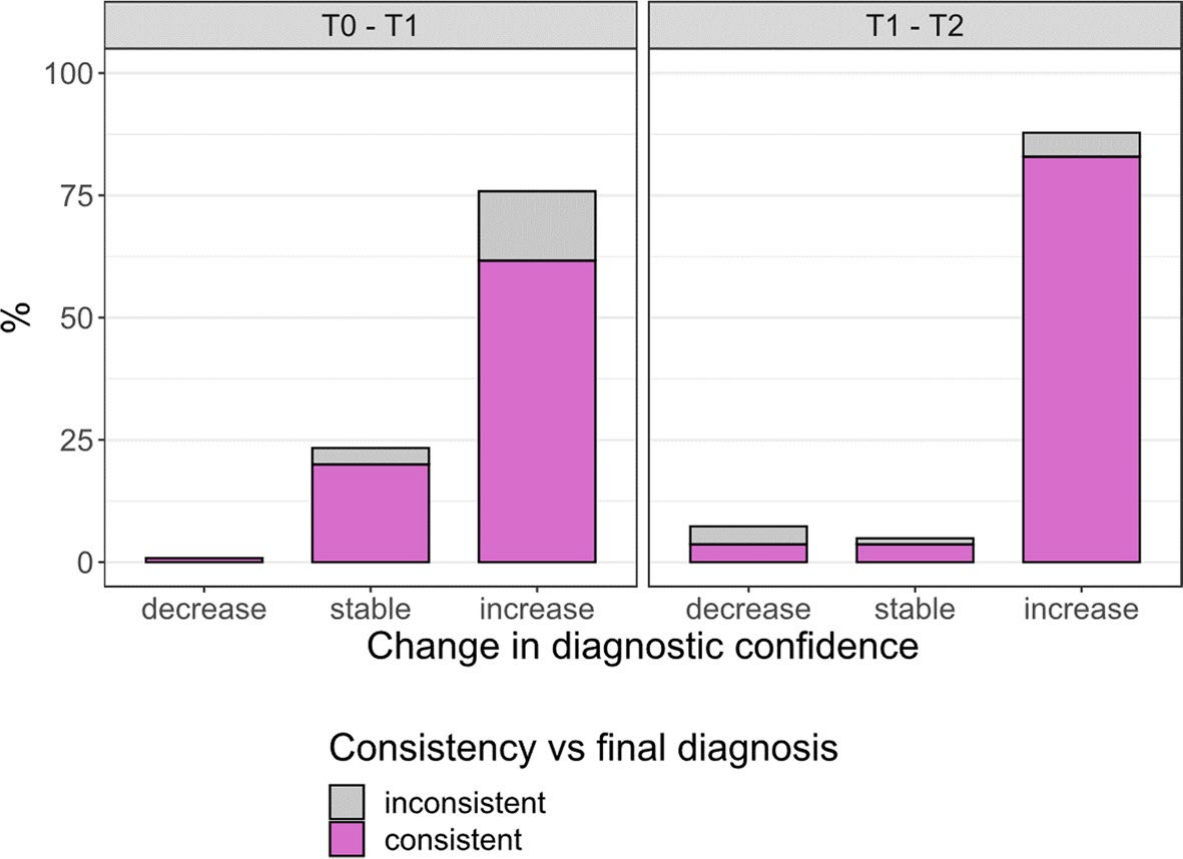
Change in diagnostic confidence in patients with confirmed diagnosis across time points, and consistency with final diagnosis (i.e., the gold standard diagnosis). Diagnostic confidence was rated on a visual numeric scale made of percentages organized spatially from 50 to 100% with incremental 5%-intervals at different time points (T0, based only on clinical reports; T1, based also on plasma biomarkers; and T2, based also on traditional biomarkers). The average diagnostic confidence of concordant raters was used for the analyses

Figure 3 illustrates the change in diagnostic confidence only in patients for whom the diagnosis was confirmed across time points (*n* = 120 from T0 to T1, and *n* = 82 from T1 to T2).

At T1, after the presentation of plasma biomarkers, diagnostic confidence increased in 76% (91/120) of cases, with a statistically significant estimated marginal mean increase of + 5% (from 78 to 83%, *p* < 0.001); while it remained stable in 23% (28/120), and decreased in < 1% (1/120) of cases. The increase in diagnostic confidence after plasma biomarkers was consistent with the final diagnosis in 81% (74/91) of cases.

At T2, after the presentation of traditional biomarkers, diagnostic confidence increased in a further 88% (72/82) of cases, with a further statistically significant estimated marginal mean increase of + 7% (from 83 to 90%, *p* < 0.001); while it remained stable in 5% (4/82), and decreased in 7% (6/82) of cases. The increase in diagnostic confidence after traditional biomarkers was consistent with the final diagnosis in 94% (68/72) of cases.

The increases in diagnostic confidence observed at T1 and T2 were not statistically different (*p* = 0.213). No differences in change in diagnostic confidence have been observed among different diagnoses (*p* = 0.353).

## Discussion

In the present study, we assessed the clinical impact of plasma biomarkers in an academic tertiary care center. Our results suggest that plasma biomarkers might support clinicians by increasing their diagnostic confidence consistently with the final diagnosis, despite a negligible impact on diagnosis. To the best of our knowledge, this is the first study investigating the diagnostic value of plasma biomarkers in a population of patients with neurodegenerative disorders.

Blood-based biomarkers are in the spotlight as they might be soon brought to clinical practice and therefore redefine and improve the diagnostic pathway of patients with suspected neurodegenerative disorders. Moreover, they might also be used to profile the risk of developing dementia in cognitively unimpaired individuals with or without genetic or lifestyle risk factors [34]. A recent review estimated that plasma biomarkers might be added to the clinicians’ armamentarium of diagnostic tests in memory clinics in the short term (3–5 years), and might be used as a screening test in primary care in the intermediate term (5–10 years), while screening of the general population is a longer-term vision [15]. In order to get ready for that, further research is needed to understand the role and performance of plasma biomarkers in clinical practice.

Our findings, although preliminary, support the future utility of plasma biomarkers on clinical grounds: the observed increased clinicians’ diagnostic confidence in most cases denoted a positive attitude towards their clinical use, and it is worth noting that such increase in diagnostic confidence was mostly consistent with the final diagnosis determined after the conclusion of the diagnostic clinical and instrumental work-up. The clinical use of plasma biomarkers is still not recommended as they are not clinically validated yet. Nevertheless, evidence on plasma biomarkers is building up fast and the field of neuroscience is rapidly evolving to accommodate their clinical implementation. The present pilot study aimed to resemble the future clinical practice where plasma biomarkers are available. Indeed, it is reasonable to assume that plasma biomarkers might be considered as a first level screening/assessment of patients with cognitive complaints, and therefore precede and be used as a gateway to traditional and more expensive or invasive biomarkers such as amyloid-PET or CSF.

In the present study, we considered a plethora of different plasma biomarkers. It has been shown that both plasma p-tau_181_ and plasma p-tau_231_are specific to AD [9, 10], and distinguish AD from FTD with very high accuracy [9]; and that plasma NfL reflects neurodegeneration (being elevated both in AD and in other neurodegenerative diseases), and discriminate FTD from psychiatric disorders [12]. Conversely, the clinical role of Aβ and GFAP might be more blurred: Aβ_42_/Aβ_40_has poor robustness and might result in misclassification [35, 36]; and GFAP, an early biomarker of astrocytosis [13], requires a more complex clinical interpretation which goes beyond the definition of pathophysiological (Aβ and p-tau) or neurodegeneration (NfL) biomarkers. Defining the most clinically and technically robust assays at the individual level may help identify which plasma biomarkers (or combination thereof) contribute to the achievement of the highest diagnostic confidence and avoid misclassification [37]. At the same time, it is important to define guidelines to facilitate clinicians’ interpretation of plasma biomarkers.

We acknowledge that the main limitation of this study is its retrospective nature. The evaluation of clinical reports does not allow the clinical observation of the patient, which is a key element of the diagnostic process. Nevertheless, our study design allowed to assess the incremental diagnostic value of plasma biomarkers in a structured way, which is not fully applicable in clinical practice where the results of the exams might not be presented always in the same order (i.e., plasma before traditional biomarkers, in this case). Second, we tested the clinical impact of plasma biomarkers in a patient population where AD is underrepresented, mostly consisting of patients with dementia due to FTLD with an already clear clinical profile at first consultation. This prevents us from assessing the full clinical potential of plasma biomarkers of AD, which is expected to be higher in a population of memory clinic patients with higher prevalence of patients at the MCI stage and with suspected AD etiology. Third, we did not consider possible confounders, such as renal failure, use of medications, or other comorbidities, which might have played a role on the performance of plasma biomarkers, thereby possibly causing misclassifications. However, all biological samples were collected with the same protocol at the first visit, and the analyses performed in a central laboratory; this allowed us to reduce the pre-analytical differences and inter-laboratory variations. Finally, we acknowledge that developing evidence suggests that p-tau_217_could have slightly greater accuracy for AD pathology [38]; however, this plasma biomarker was not available for the present study.

To conclude, our findings suggest that plasma biomarkers may support clinicians by increasing their diagnostic confidence, despite a negligible impact on diagnosis. The present study lays the groundwork for prospective and possibly randomized controlled studies assessing the role and full clinical potential of plasma biomarkers in a memory clinic setting.

### Supplementary Information

Supplementary Material 1. 

## Data Availability

No datasets were generated or analysed during the current study.
