# Supplementary material for: Plasma biomarkers increase diagnostic confidence in patients with Alzheimer’s disease or frontotemporal lobar degeneration
Source: Alzheimers Res Ther. 2024 May 11;16:107. doi: 10.1186/s13195-024-01474-z (PMC11088144; doi:10.1186/s13195-024-01474-z)
Supplement: Supplementary file 1 — Supplementary Material 1. [file 13195_2024_1474_MOESM1_ESM.docx]

**Plasma biomarkers increase diagnostic confidence in patients with Alzheimer’s disease or Frontotemporal Lobar Degeneration**

Daniele Altomare, PhD; Ilenia Libri, MD; Antonella Alberici, MD; Jasmine Rivolta, MS; Alessandro Padovani, MD, PhD; Nicholas J. Ashton, PhD; Henrik Zetterberg, MD, PhD; Kaj Blennow, MD, PhD; Barbara Borroni, MD

**Content**

**e2.1**. Material and preparatory activities.

**eTable**. Descriptive statistics of plasma biomarkers disaggregating by final diagnosis.

**eFigure**. Distribution of plasma biomarkers disaggregating by final diagnosis.

**e2.1. Material and preparatory activities**

The preparatory activities and material described below were necessary to ensure the reproducibility of the present study.

Since the research field of blood-based biomarkers is rapidly evolving, all raters underwent a training session (lasting about 60 minutes) before the beginning of the study, consisting in the revision and discussion of the most relevant and recent papers on this topic. During the assessment of the clinical reports, the raters had two forms: the first form reported the description of a sample of 27 cognitively unimpaired individuals (median±IQR age: 48±26 years; gender: 44% (12/27) of males) consisting in the minimum value, median and IQR, mean and SD, and maximum value as well as the distribution (displayed as scatterplots and boxplots of the plasma values converted into Z-scores) of each plasma biomarker; the second was a rating questionnaire with all the information they were asked to provide at the different timepoints. Finally, the raters were asked to fill in a questionnaire (already used in a previous study on the clinical use of Alzheimer’s disease biomarkers in patients with mild cognitive impairment^1^) on raters’ profiling and on the use of biomarkers in clinical practice. Specifically, raters were asked about their belief/opinion about the pathogenic role of amyloid and tau in Alzheimer’s disease pathology and symptoms using a 0-10 Likert scale (0-4: prevalent role of amyloid, 5: amyloid and tau have the same relevance or are not relevant, 6-10: prevalent role of tau).

Both raters attributed a prevalent pathogenic role to amyloid in Alzheimer’s disease pathology and symptoms (scores 8 and 7, respectively).

**eTable** (part A). Descriptive statistics of plasma biomarkers disaggregating by final diagnosis.

| **Plasma**  **biomarker** | **Values** | **CU**  **n=27** | **AD**  **n=29** | **FTLD** | |
| --- | --- | --- | --- | --- | --- |
|  |  |  |  | **FTD**  **n=80** | **CBS/PSP**  **n=13** |
| **Aβ_42_** | Min | 0.18 | 0.54 | 0.29 | 1.07 |
|  | Median | 5.30 | 2.92 | 5.21 | 3.91 |
|  | IQR | 3.85 | 2.66 | 3.91 | 3.58 |
|  | Mean | 5.13 | 3.65 | 4.93 | 4.37 |
|  | SD | 2.70 | 2.77 | 3.07 | 2.50 |
|  | Max | 9.99 | 11.50 | 14.60 | 8.51 |
| **Aβ_40_** | Min | 1.73 | 0.54 | 1.12 | 7.28 |
|  | Median | 60.80 | 36.20 | 61.95 | 51.80 |
|  | IQR | 55.35 | 59.52 | 55.12 | 61.80 |
|  | Mean | 53.23 | 47.66 | 59.72 | 51.17 |
|  | SD | 33.54 | 46.10 | 45.32 | 35.40 |
|  | Max | 123.00 | 175.00 | 221.00 | 117.00 |
| **Aβ_42_/Aβ_40_** | Min | 0.06 | 0.04 | 0.03 | 0.03 |
|  | Median | 0.09 | 0.07 | 0.09 | 0.08 |
|  | IQR | 0.02 | 0.08 | 0.03 | 0.06 |
|  | Mean | 0.09 | 0.13 | 0.12 | 0.09 |
|  | SD | 0.02 | 0.11 | 0.10 | 0.04 |
|  | Max | 0.12 | 0.52 | 0.57 | 0.15 |
| **p-tau_181_** | Min | 0.45 | 0.36 | 0.01 | 0.92 |
|  | Median | 3.81 | 10.92 | 5.17 | 5.34 |
|  | IQR | 4.24 | 12.83 | 7.92 | 6.52 |
|  | Mean | 4.25 | 13.14 | 8.93 | 5.92 |
|  | SD | 2.55 | 10.92 | 10.57 | 4.16 |
|  | Max | 8.97 | 43.11 | 61.35 | 13.07 |

**eTable** (part B). Descriptive statistics of plasma biomarkers disaggregating by final diagnosis.

| **Plasma**  **biomarker** | **Values** | **CU**  **n=27** | **AD**  **n=29** | **FTLD** | |
| --- | --- | --- | --- | --- | --- |
|  |  |  |  | **FTD**  **n=80** | **CBS/PSP**  **n=13** |
| **p-tau_231_** | Min | 0.01 | 0.01 | 0.01 | 0.13 |
|  | Median | 4.45 | 3.67 | 4.07 | 2.85 |
|  | IQR | 4.73 | 9.26 | 5.98 | 5.14 |
|  | Mean | 4.20 | 5.74 | 4.86 | 3.60 |
|  | SD | 3.10 | 6.60 | 4.60 | 3.32 |
|  | Max | 12.15 | 24.90 | 18.97 | 9.26 |
| **NfL** | Min | 3.02 | 7.52 | 3.22 | 11.10 |
|  | Median | 6.71 | 23.10 | 28.00 | 28.30 |
|  | IQR | 6.81 | 9.60 | 26.05 | 16.90 |
|  | Mean | 8.42 | 26.82 | 41.46 | 42.10 |
|  | SD | 4.44 | 24.69 | 43.72 | 51.84 |
|  | Max | 20.40 | 143.00 | 271.00 | 209.00 |
| **GFAP** | Min | 24.68 | 45.63 | 29.95 | 39.36 |
|  | Median | 66.29 | 208.89 | 134.80 | 123.34 |
|  | IQR | 48.65 | 112.60 | 121.28 | 66.22 |
|  | Mean | 71.39 | 225.33 | 159.79 | 143.42 |
|  | SD | 31.68 | 163.90 | 100.83 | 102.01 |
|  | Max | 139.03 | 957.15 | 529.84 | 450.05 |

AD: Alzheimer’s disease. CBS: corticobasal syndrome. CU: cognitively unimpaired. FTD: frontotemporal dementia. FTLD: frontotemporal lobar degeneration. PSP: progressive supranuclear palsy.

The values of CU were reported to the study raters as a reference to assess plasma biomarkers in patients.

**eFigure**. Distribution of plasma biomarkers disaggregating by final diagnosis.


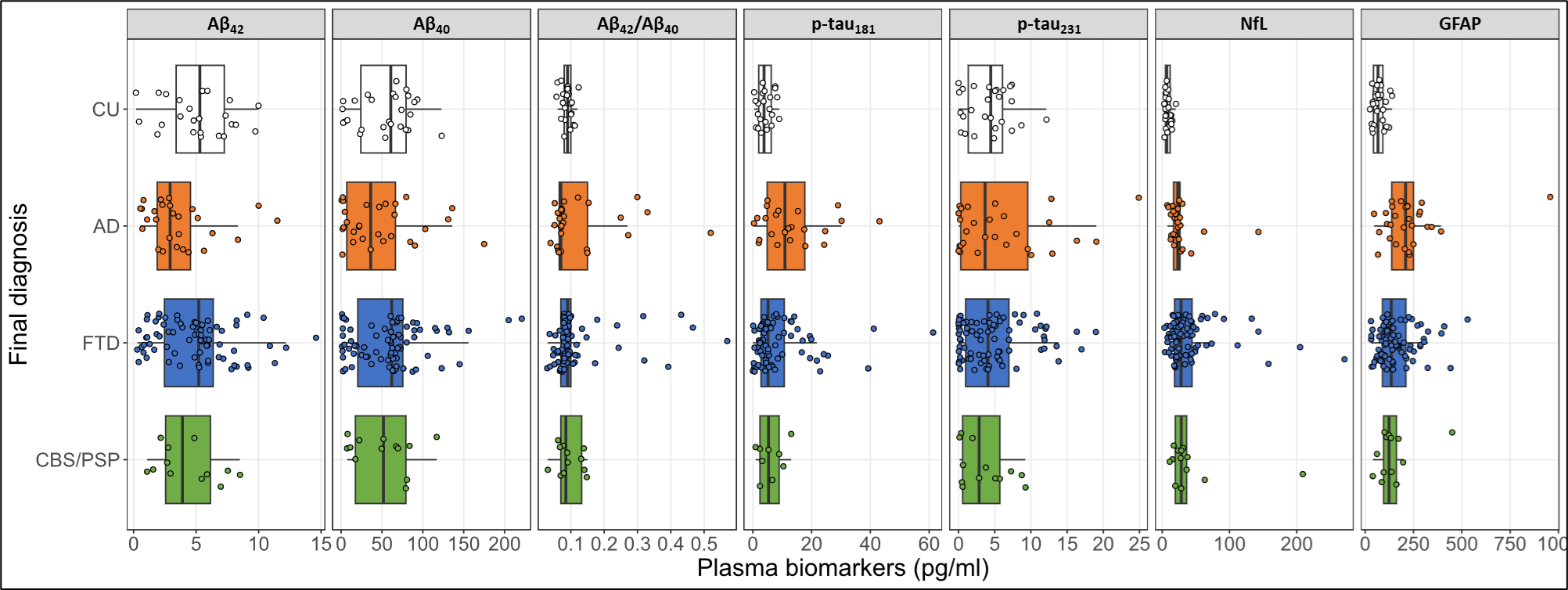


AD: Alzheimer’s disease. CBS: corticobasal syndrome. CU: cognitively unimpaired. FTD: frontotemporal dementia. PSP: progressive supranuclear palsy.

**REFERENCE**

1 Caprioglio C, Garibotto V, Jessen F, *et al.* The Clinical Use of Alzheimer’s Disease Biomarkers in Patients with Mild Cognitive Impairment: A European Alzheimer’s Disease Consortium Survey. *J Alzheimers Dis* 2022; **89**: 535–51.
